# Assessment of inhaler techniques employed by patients with respiratory diseases in southern Brazil: a population-based study*

**DOI:** 10.1590/S1806-37132014000500007

**Published:** 2014

**Authors:** Paula Duarte de Oliveira, Ana Maria Baptista Menezes, Andréa Dâmaso Bertoldi, Fernando César Wehrmeister, Silvia Elaine Cardozo Macedo

**Affiliations:** Graduate Program in Epidemiology, Federal University of Pelotas, Pelotas, Brazil; Graduate Program in Epidemiology, Federal University of Pelotas, Pelotas, Brazil; Graduate Program in Epidemiology, Federal University of Pelotas, Pelotas, Brazil; Graduate Program in Epidemiology, Federal University of Pelotas, Pelotas, Brazil; Graduate Program in Epidemiology, Federal University of Pelotas, Pelotas, Brazil

**Keywords:** Asthma, Pulmonary disease, chronic obstructive, Dry powder inhalers, Metered dose inhalers

## Abstract

**OBJECTIVE:**

To identify incorrect inhaler techniques employed by patients with respiratory diseases in southern Brazil and to profile the individuals who make such errors.

**METHODS:**

This was a population-based, cross-sectional study involving subjects ≥ 10 years of age using metered dose inhalers (MDIs) or dry powder inhalers (DPIs) in 1,722 households in the city of Pelotas, Brazil.

**RESULTS:**

We included 110 subjects, who collectively used 94 MDIs and 49 DPIs. The most common errors in the use of MDIs and DPIs were not exhaling prior to inhalation (66% and 47%, respectively), not performing a breath-hold after inhalation (29% and 25%), and not shaking the MDI prior to use (21%). Individuals ≥ 60 years of age more often made such errors. Among the demonstrations of the use of MDIs and DPIs, at least one error was made in 72% and 51%, respectively. Overall, there were errors made in all steps in 11% of the demonstrations, whereas there were no errors made in 13%.Among the individuals who made at least one error, the proportion of those with a low level of education was significantly greater than was that of those with a higher level of education, for MDIs (85% vs. 60%; p = 0.018) and for DPIs (81% vs. 35%; p = 0.010).

**CONCLUSIONS:**

In this sample, the most common errors in the use of inhalers were not exhaling prior to inhalation, not performing a breath-hold after inhalation, and not shaking the MDI prior to use. Special attention should be given to education regarding inhaler techniques for patients of lower socioeconomic status and with less formal education, as well as for those of advanced age, because those populations are at a greater risk of committing errors in their use of inhalers.

## Introduction

Controlled-dose inhalers are the primary means of delivering treatment for respiratory diseases, such as asthma and COPD, both of which have a significant prevalence worldwide.^(^ 1 ^,^ 2 ^)^

The inclusion of these inhalers in the routine treatment of respiratory diseases occurred in the 1950s, with the development of the first metered dose inhalers (MDIs). Subsequently, in the 1990s, dry powder inhalers (DPIs) were developed. ^(^ 3 ^-^ 5 ^)^ The drugs delivered through such inhalers have bronchodilating and/or anti-inflammatory activity. ^(^ 1 ^,^ 3 ^,^ 6 ^)^ Their deposition directly in the target organ has advantages, such as a reduction in systemic adverse effects and a rapid reduction in symptoms. ^(^ 4 ^,^ 6 ^,^ 7 ^)^

Each type of inhaler device has specific characteristics that are relevant to its correct use. The choice of inhaler to be prescribed depends on a number of factors, ranging from patient familiarity with the technique for use of a given inhaler and the degree of deposition in the airways to cost-benefit ratio, portability, and patient cognition.^(^ 8 ^-^ 11 ^)^

A factor that has been documented as one of the major contributors to poor asthma and COPD control, resulting in an increased number of emergency room visits and preventable hospitalizations, is incorrect inhaler technique.^(^ 12 ^,^ 13 ^)^

Therefore, the objective of the present study was to identify, through a checklist of steps for performing the inhaler technique, the most common errors made by patients and to profile the individuals who make such errors.

## Methods

The present open-label, uncontrolled, cross-sectional, observational study was part of a population-based, cross-sectional study conducted in 2012 and involving subjects ≥ 10 years of age in 1,722 households in the city of Pelotas, Brazil.^(^ 14 ^)^

Those who reported having received a physician diagnosis of asthma, bronchitis, or emphysema and who used controlled-dose inhalers were invited to participate in the present study, which was conducted between May and September of 2012. Inhaler technique was assessed, and a small questionnaire, which was additional to the main study, was administered to collect information on inhaler use. Those who required assistance from others to use the inhaler or who used the MDI with a spacer and a mask were excluded because they would not complete the steps included in the checklist.

The variables collected in the main study that were also analyzed in the present study were gender, age, level of education (in years of schooling), and socioeconomic status, as determined on the basis of the *Indicador Econômico Nacional* (IEN, National Economic Indicator), categorized into tertiles. Participants were asked who had recommended using an inhaler (pulmonologists, general practitioners, other specialists, or lay people), whether the physician had provided inhaler technique demonstration to them at the time of prescription (yes/no), the frequency of inhaler use (chronic or only during exacerbations), and where the inhaler had been acquired (at an ordinary pharmacy, through the Brazilian Popular Pharmacy program,^(^ 15 ^)^ through the public health care system, or others).

Inhaler technique was assessed using a checklist established for each inhaler model on the basis of the Third Brazilian Consensus on Asthma Management recommendations for the use of inhalers.^(^ 8 ^)^ Questionnaire completion and technique assessment were conducted in the participant's household by two trained interviewers. Observation of technique consisted of asking inhaler users to demonstrate how they took their treatment by using their own inhaler or a placebo device provided at the time of the interview. After completion of the checklist, individuals received instructions regarding the steps that needed to be corrected. Subsequently, data were entered in duplicate into EpiData, version 3.1 (EpiData Association, Odense, Denmark) and checked for inconsistencies.

Patient performance of inhaler technique was assessed in different ways: 1) main inhalation steps: pre-inhalation (including dose preparation); inhalation (from breathing out before breathing in to drug inhalation); and post-inhalation (breath-holding at the end of inhalation and repeating the procedure if necessary). This information allowed the creation of dichotomous variables; each fully completed step was categorized as "yes/no", according to the specific characteristics of each inhaler; 2) occurrence of at least one error as per the checklist items, categorized as "yes/no"; and 3) proportion of committed errors as per the checklist items, which originated a continuous variable. The last two assessment categories included from breathing out before inhalation to breath-holding at the end of inhalation.

Results were described by using inhalers as the unit of analysis, because each inhaler has particular characteristics of use, even when used by the same individual. We used absolute and relative frequencies and their 95% CIs. Bivariate analyses were performed by using the chi-square test for heterogeneity and the test for linear trends for dichotomous outcomes, as well as the Kruskal-Wallis test for non-normally distributed numerical outcomes. We used the STATA statistical software package, version 12.0 (StataCorp LP, College Station, TX, USA).

Similarly to the main study, this stage of the project was approved by the Research Ethics Committee of the Federal University of Pelotas School of Medicine (Protocol no. 77/11; December 1, 2011), and substudy participants or their legal guardians gave written informed consent.

## Results

**Figure 1:**
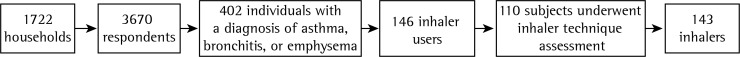
Flowchart of sample composition.

Figure 1 describes the main study sample and the substudy sample. The substudy sample consisted of 110 individuals who agreed to undergo inhaler technique assessment. In total, 143 inhalers (94 MDIs and 49 DPIs) were used. Of the 146 inhaler users identified in the main study, 21 (14.4%) declined to participate or were not located during the course of the substudy, 8 (5.5%) were excluded because they were unable to perform the inhaler technique without assistance, and 7 (4.8%) were excluded because they used a spacer with a mask.

Of the 49 DPIs used, 40 were capsule inhalers, 7 were Diskus inhalers, and 2 were Turbuhalers. Other models were not used among the study participants, except for Respimat inhalers, which were used by 3 individuals. This model was excluded from the analyses because the technique for using it has special characteristics and because of the small number of observations.

Most inhalers were acquired at an ordinary pharmacy (77%), whereas some were acquired through the public health care system (11%) and some were acquired through the Brazilian Popular Pharmacy program (10%). The remaining inhalers were acquired otherwise, such as through donations or by lawsuit.

The most common errors in the use of MDIs and DPIs were not exhaling prior to inhalation (an error made by more than half of the total sample) and failure to breath-hold after inhalation (Table 1). In the assessment of DPI technique, not shaking the inhaler and not waiting 15-30 seconds prior to a second inhalation (among those who took more than one dose) were also common. The proportions of all errors, as per the checklist items, were higher in the 60-or-older age group, except for the error of not exhaling prior to inhalation via an MDI, which showed relative homogeneity among all age groups.

**Table 1:** Errors in metered dose and dry powder inhaler techniques, i.e., inhaler technique steps that were performed incorrectly, in the total sample and by age group (N = 143 inhalers).a

Error	Total sample	Age group		
		10-19 years	20-59 years	60 years or older
Metered dose inhaler	(n = 94)	(n = 24)	(n = 61)	(n = 9)
Not shaking the inhaler	20 (21.3)	6 (25.0)	11 (18.0)	3 (33.3)
Not holding the mouthpiece vertically 4-5 cm away from the mouth or between the lips	3 (3.2)	1 (4.2)	2 (3.3)	0 (0.0)
Not keeping the mouth open	2 (2.1)	1 (4.2)	1 (1.6)	0 (0.0)
Not exhaling normally	62 (66.0)	16 (66.7)	41 (67.2)	5 (66.0)
Not actuating the inhaler at the start of a slow and deep inhalation	7 (7.5)	1 (4.2)	4 (6.6)	2 (22.2)
Failure to breath-hold for at least 10 seconds after inhalation	27 (28.7)	7 (29.2)	16 (26.2)	4 (44.4)
Not waiting 15-30 seconds prior to each actuation^b^	45 (57.7)	11 (55.0)	26 (54.2)	8 (88.9)
Dry powder inhaler	(n = 49)	(n = 4)	(n = 29)	(n = 16)
Error in dose preparation (all models)	3 (6.1)	0 (0.0)	0 (0.0)	3 (18.8)
Not exhaling normally	23 (46.9)	1 (25.0)	12 (41.4)	10 (62.5)
Not placing the inhaler in the mouth	1 (2.0)	0 (0.0)	0 (0.0)	1 (6.3)
Not inhaling as fast and as deeply as possible	3 (6.1)	0 (0.0)	0 (0.0)	3 (18.7)
Failure to breath-hold for 10 seconds after inhalation	12 (24.5)	0 (0.0)	7 (24.1)	5 (31.3)
Single-dose dry powder inhaler: not inhaling again, more deeply than before, if there is powder left in the capsule^c^	7 (17.5)	1 (25.0)	2 (8.7)	4 (30.7)

Table 2 shows the description of inhaler use in the sample by sociodemographic and prescription characteristics, as well as the distribution of inhaler technique errors by those characteristics. Among MDI and DPI users, 72% and 51%, respectively, made some error during drug inhalation. The mean proportion of errors as per the checklist items was 21%. Those with a low level of education (up to 8 years of schooling) made more errors in the use of the two types of inhaler than did those with a higher level of education. Likewise, there were significant differences among the IEN tertiles in terms of the occurrence of any MDI technique error and the mean proportion of errors, with this proportion being greatest among the most economically disadvantaged. The differences in performance of inhaler technique among the other variables showed no statistical significance.

**Table 2:** Description of inhaler use, by demographic variables, socioeconomic variables, source of recommendation, technique demonstration, and frequency of use (N = 143 inhalers).

Variable	Metered dose inhaler			Dry powder inhaler			Mean percentage of errors (SE)^†^	p
	n (%)	% of any error in technique (95% CI)	p	n (%)	% of any error in technique (95% CI)	p		
Gender								
Male	29 (30.8)	65.5 (47.7-83.4)	0.323	16 (32.6)	43.8 (18.0-69.5)	0.478	19.1 (21.5)	0.379
Female	65 (69.2)	75.4 (64.7-86.1)		33 (67.4)	54.5 (36.8-72.2)		21.8 (20.0)	
Age, years								
10-19	24 (25.5)	79.2 (62.4-96.0)	0.376*	4 (8.2)	25.0 (0.0-75.3)	0.219*	19.5 (17.7)	0.399
20-39	32 (34.0)	71.9 (55.8-87.9)		10 (20.4)	50.0 (16.5-83.5)		17.5 (15.0)	
40-59	29 (30.9)	69.0 (51.6-86.3)		19 (38.8)	47.4 (23.7-71.0)		21.3 (22.2)	
60 or older	9 (9.6)	66.7 (33.5-99.8)		16 (32.7)	62.5 (37.4-87.6)		27.8 (26.4)	
Level of education, years								
0-8	46 (48.9)	84.8 (74.1-95.4)	0.018*	16 (32.7)	81.3 (61.0-100.0)	0.010*	27.5 (18.0)	< 0.001
9-11	28 (29.8)	60.7 (42.0-79.4)		16 (32.7)	37.5 (12.4-62.6)		16.7 (21.2)	
12 or more	20 (21.3)	60.0 (37.7-82.3)		17 (34.7)	35.3 (11.3-59.3)		15.0 (20.6)	
IEN, tertiles^a^								
1 (the poorest)	25 (26.9)	88.0 (74.8-100.0)	0.044*	11 (22.5)	72.7 (44.4-100.0)	0.080*	27.2 (17.4)	0.017
2	38 (40.9)	71.1 (56.2-85.9)		22 (44.9)	50.0 (28.1-71.9)		21.3 (22.0)	
3 (the richest)	30 (32.3)	63.3 (45.6-81.1)		16 (32.7)	37.5 (12.4-62.6)		16.0 (19.5)	
Recommendation for inhaler use								
Pulmonologist	35 (37.2)	60.0 (43.3-76.7)	0.090	35 (72.9)	44.4 (27.6-61.3)	0.125	17.3 (20.8)	0.073
General practitioner/another specialist	49 (52.1)	81.6 (70.5-92.7)		13 (27.1)	69.2 (42.4-96.0)		25.3 (20.0)	
Non-physician	10 (10.6)	70.0 (39.7-100.0)		0 (0.0)	-		20.0 (16.3)	
Received a demonstration by a physician								
Yes	52 (55.3)	69.2 (56.4-82.1)	0.375	36 (73.5)	43.2 (26.6-59.8)	0.056	18.4 (20.3)	0.112
No	32 (34.0)	78.1 (63.4-92.9)		13 (26.5)	75.0 (48.7-100.0)		26.3 (21.0)	
Frequency of use								
Chronic	23 (24.5)	69.6 (50.1-89.0)	0.732	31 (63.3)	54.8 (36.5-73.1)	0.483	23.0 (21.3)	0.216
Attacks	71 (75.5)	73.2 (62.7-83.7)		18 (36.7)	44.4 (20.2-68.7)		19.7 (19.9)	
TOTAL	94 (100)	72.4 (63.1-81.6)		49 (100)	51.0 (36.5-65.5)		20.9 (20.4)	

IEN: Indicador Econômico Nacional (National Economic Indicator). an = 142. p values determined with the chi-square test for heterogeneity, except otherwise indicated.trend. *Chi-square test for trend. †Kruskal-Wallis test

**Figure 2:**
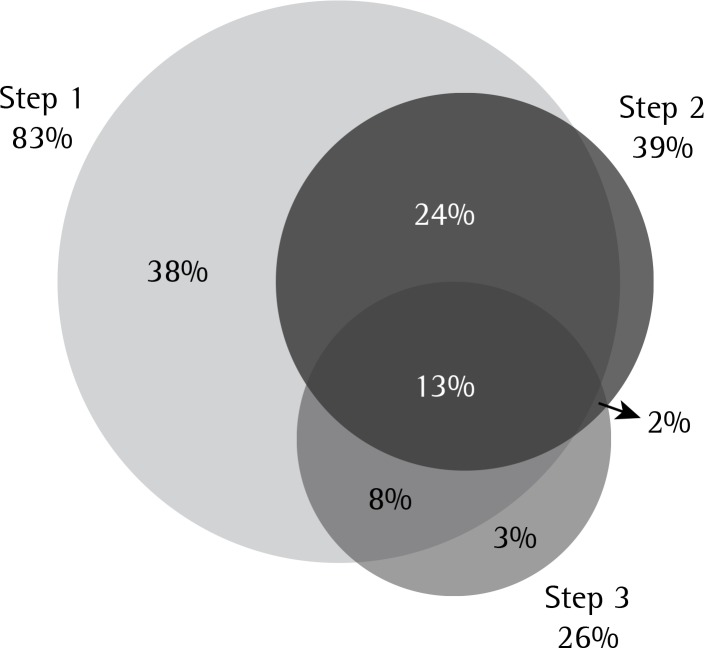
Venn diagram showing the proportions of correct demonstrations by inhaler use step (N = 143 inhalers). Step 1, pre-inhalation, with correct dose preparation. Step 2: exhalation and correct positioning of the inhaler at the mouth until the end of inhalation. Step 3: correct completion of the technique. In 16 demonstrations (11%), there were errors made in all steps.

Figure 2 shows a Venn diagram of the inhaler technique at three steps: pre-inhalation; inhalation; and post-inhalation. There were no errors made in only 13% of the demonstrations, whereas there were errors made in all steps in 11% of the demonstrations during the completion of the checklist.

## Discussion

The present study describes patient difficulties in using inhalers in the treatment of asthma and COPD. Being a population-based study, it makes it possible to establish a general profile of users of this type of medication, whether they are patients who are being followed at a health care facility, they are those who haven't had a medical visit for a long time, or they are those to whom inhaler use was recommended by lay people.

One limitation of the present study is that we did not assess issues related to the severity/staging of respiratory diseases or the appropriateness of the recommended inhalation treatment, nor did we investigate associations of that treatment with control of lung diseases, which could reinforce the need for proper inhaler technique. In addition, because of the limited sample size, there was no statistical power to detect some associations between technique performance and certain characteristics of individuals. It was only possible to detect significant differences (p ≤ 0.05) for prevalence ratios equal to or greater than 1.5.

Another possible limiting factor of our results is information bias. Although inhaler users were not previously informed that their technique would be assessed, they may have performed the technique more correctly because they were being watched, that performance being different from their routine performance and the number of detected errors being smaller. Also, it was not possible to objectively determine whether the inspiratory flow from the DPIs achieved the minimum speed recommended for the different inhalers.

The proportion of occurrence of any inhaler technique error, as per the checklist, relative to the number of inhalers tested was smaller than expected given previous findings in the literature: in one study, although patients reported knowing the proper inhaler technique, approximately 90% made some error.^(^ 16 ^)^ In addition, in a telephone survey, 77 of 87 respondents reported that their technique had never been checked by a health care professional, and, of 26 patients selected for a demonstration, none achieved satisfactory performance.^(^ 17 ^)^ In contrast, a study conducted in the state of Bahia, Brazil, reported that more than half of the individuals studied showed good inhaler technique for all inhaler models; however, the sample consisted of individuals who received follow-up and underwent inhaler technique assessment periodically, and the criterion for classification of patient performance of the technique as good was 75% of steps correctly completed or more.^(^ 18 ^)^

The characteristics of patients requiring inhaler use also deserve significant attention at the time of prescription. Previous studies have reported that elderly patients make more errors because they have cognitive changes, among other factors. ^(^ 12 ^,^ 19 ^,^ 20 ^)^ In our study, the proportion of errors was greater among patients in the 60-or-older age group; however, we could not detect a significant difference in their inhaler technique relative to that of patients in the younger age groups. The 60-or-older age group was the smallest in our sample, and this was possibly the factor that prevented the detection of significant differences relative to the technique used by younger patients. One explanation for the low number of participants in this advanced age group would be the lower number of MDI users, even though this group has a significant number of subjects who report having respiratory diseases.^(^ 14 ^)^ Many may not have adapted to this type of inhaler and prefer to use nebulization, which is indicated for those who are too cognitively impaired to use other inhaled drug delivery systems^(^ 1 ^,^ 2 ^)^; in addition, one exclusion criterion of the present study was requiring assistance from others to use the inhaler or using the MDI with a spacer and a mask, resources often used in this age group. According to a previous study,^(^ 21 ^)^ nebulizer users are of advanced age, have respiratory conditions that are more severe, and have great difficulty in using MDIs.

Our results showed a significant linear trend toward a higher frequency of errors among those of lower socioeconomic status (except for DPIs, with p > 0.05, but with a trend in the same direction) and with a lower level of education. These results are consistent with previous findings. ^(^ 12 ^,^ 19 ^)^ Level of education and socioeconomic status are similar indicators and show the importance of the level of education not only as a facilitator in the understanding of the technique itself, but also in the understanding of the disease,^(^ 13 ^)^ generally improving treatment adherence. These findings suggest the need for implementing educational measures regarding the disease and the inhaler technique, especially among those who are socioeconomically disadvantaged.

The most common errors regarding the use of MDIs described in the literature are as follows: not exhaling prior to inhalation and failure to breath-hold after inhalation; incorrect positioning of the inhaler; and failure to inhale forcefully and deeply.^(^ 16 ^,^ 22 ^)^ In our study, the presence of most of these errors was significant.

The number of errors was greater for MDIs than for DPIs. This fact has been documented in the literature, such as in a study^(^ 23 ^)^ that reported that the proportion of individuals who made at least one inhaler technique error, as well as the proportion of errors considered of greatest importance to the effectiveness of treatment, was greater for MDIs than for DPIs.

Many individuals also do not shake the MDI before using it. At the time the study was conducted, the technique checklist was developed on the basis of a Brazilian consensus,^(^ 8 ^)^ and this step was still considered mandatory for all MDIs. However, according to more recent guidelines, depending on the type of drug formulation (solution or suspension), not all MDIs always need to be shaken^(^ 1 ^)^; nevertheless, most guidelines on inhaler technique maintain the recommendation that the device be shaken before use,^(^ 24 ^)^ because it is known that failure to perform this step can reduce aerosol dose delivery by up to 36%.^(^ 25 ^)^

Another difference between MDI techniques as per that consensus report^(^ 8 ^)^ and those as per the current guidelines on the management of asthma^(^ 1 ^)^ was the removal of chlorofluorocarbon from the formulations and its replacement with hydrofluoroalkane (HFA). Therefore, the technique was facilitated-HFA allows a longer puff duration, reducing the need for fine coordination of actuation and inhalation.^(^ 1 ^)^ Although problems in inhalation-actuation synchrony are well documented,^(^ 25 ^)^ problems in this step were hardly detected, probably because of the eligibility criteria for participation in the study.

Another error documented in the literature is inadequate distance between the inhaler and the patient's lips^(^ 16 ^)^; however, we did not consider the fact that many subjects place the inhaler between their lips at the time of inhalation to represent an error. According to the most recent guidelines, it is acceptable to perform the inhaler technique this way, because it reduces evaporation of gas, increasing pulmonary deposition and reducing the risk of the spray hitting the perioral region.^(^ 1 ^)^

Another failure in technique detected in our study, as well as in previous studies,^(^ 16 ^,^ 22 ^)^ is the fact that individuals do not exhale prior to inhalation. According to a review article whose primary objective was to determine the importance of this one step, it is recommended to exhale gently to functional residual capacity or residual volume, thereby optimizing the effectiveness of the inhaler technique.^(^ 26 ^)^ Breath-holding for 10 seconds is also among the recommendations for optimal pulmonary drug deposition for all models of inhalers. However, for MDIs, it is no longer recommended to wait 30 seconds to take another dose, as in the consensus report used in the development of our checklist.^(^ 1 ^,^ 8 ^,^ 24 ^)^

In our study, the proportion of errors was greater among individuals who did not receive a demonstration of correct inhaler use at the time of prescription than among those who did, although this result did not reach a statistically significant difference. This factor has been documented as a contributor to unsatisfactory inhaler technique.^(^ 27 ^)^ It should be highlighted that many health care professionals do not have adequate knowledge regarding the use of DPIs and MDIs, which contributes to the great proportion of subjects who take their inhaled drugs incorrectly, even if they previously received a demonstration of inhaler use.^(^ 28 ^)^ It is of note that, in addition to training at the time of prescription, patients should receive periodic counseling throughout the treatment, because the correct technique is usually forgotten over time.^(^ 22 ^,^ 23 ^)^

In an intervention conducted by pharmacists in Germany, the inhaler technique of 757 patients with a diagnosis of asthma or COPD was assessed and recorded using a checklist, with patients receiving instructions and their errors being corrected at the first appointment. Approximately 80% made some error at baseline. One month after the initial assessment, a second demonstration was requested, and that proportion dropped to 28.3%.^(^ 29 ^)^ Therefore, educational activities, even if they are sporadic, can be beneficial to inhaler users. 

Counseling of patients and their caregivers, performed by health care professionals, plays a key role in inhaler use so as to minimize errors and optimize treatment.^(^ 9 ^,^ 17 ^,^ 22 ^,^ 26 ^)^ Poor inhaler technique is a risk factor for poor control of respiratory diseases,^(^ 12 ^,^ 13 ^)^ being associated with poor treatment adherence.^(^ 30 ^)^ It is noteworthy, however, that this is a modifiable risk factor, and some findings of the present study can act as reference points in the inhaler technique to be targeted for improvement , as well as allowing the identification of the profile of those patients who will potentially require further clarification regarding inhaler use.

We therefore conclude that the most common errors made by patients when using inhalers are not exhaling prior to inhalation and failure to breath-hold after inhalation, for MDIs and DPIs. Special attention should be given to patients of lower socioeconomic status, because they are at a greater risk of committing errors in their use of DPIs, as well as to those in advanced age groups, because they make a greater proportion of inhaler technique errors.
