# Supplementary material for: Assessment of inhaler techniques employed by patients with respiratory diseases in southern Brazil: a population-based study
Source: J Bras Pneumol. 2014 Sep-Oct;40(5):513–20. doi: 10.1590/S1806-37132014000500007 (PMC4263332; doi:10.1590/S1806-37132014000500007)
Supplement: Translation

# Translation: Avaliação da técnica de utilização de dispositivos inalatórios no tratamento de doenças respiratórias no sul do Brasil: estudo de base populacional*

**Authors:** Paula Duarte de Oliveira, Ana Maria Baptista Menezes, Andréa Dâmaso Bertoldi, Fernando César Wehrmeister, Silvia Elaine Cardozo Macedo

**Affiliations:** Programa de Pós-Graduação em Epidemiologia, Universidade Federal de Pelotas, Pelotas (RS) Brasil; Programa de Pós-Graduação em Epidemiologia, Universidade Federal de Pelotas, Pelotas (RS) Brasil; Programa de Pós-Graduação em Epidemiologia, Universidade Federal de Pelotas, Pelotas (RS) Brasil; Programa de Pós-Graduação em Epidemiologia, Universidade Federal de Pelotas, Pelotas (RS) Brasil; Departamento de Clínica Médica, Universidade Federal de Pelotas, Pelotas (RS) Brasil

**Keywords:** Asma, Doença pulmonar obstrutiva crônica, Inaladores de pó seco, Inaladores dosimetrados

## Abstract

**OBJETIVO:**

Conhecer os erros na técnica de uso de dispositivos inalatórios empregada por pacientes com doenças respiratórias no sul do Brasil e o perfil daqueles que possuem dificuldades em realizá-la.

**MÉTODOS:**

Estudo transversal, de base populacional, com indivíduos com idade ≥ 10 anos e em uso de inaladores pressurizados (IPrs) ou inaladores de pó (IP) em 1.722 domicílios de Pelotas (RS).

**RESULTADOS:**

Foram incluídos 110 indivíduos que utilizavam 94 IPrs e 49 IP. Os principais erros no uso dos IPrs e IP foram não expirar antes da inalação (66% e 47%, respectivamente), não fazer uma pausa inspiratória após a inalação (29% e 25%) e não agitar o IPr antes do uso (21%). Os indivíduos com idade ≥ 60 anos mais frequentemente cometeram erros. Das demonstrações de uso do IPr e IP, respectivamente, 72% e 51% apresentaram ao menos um erro, enquanto 13% das demonstrações foram plenamente corretas e 11% apresentaram erros em todas as fases. A proporção de indivíduos com menor nível de escolaridade que cometeram ao menos um erro foi significativamente maior do que a daqueles com maior nível de escolaridade tanto no uso de IPrs (85% vs. 60%; p = 0,018) quanto no de IPs (81% vs. 35%; p = 0,010).

**CONCLUSÕES:**

Nesta amostra, os principais erros cometidos no uso dos inaladores foram não realizar a expiração antes da inalação, não fazer a pausa inspiratória após a inalação e não agitar o IPr. Pacientes com menor nível socioeconômico e educacional, assim como aqueles com idade avançada, merecem especial atenção na educação sobre a realização da técnica inalatória, pois apresentam um maior risco de cometer erros durante o uso dos inaladores.

## Introdução

Os dispositivos inalatórios de dose controlada constituem-se no principal meio de administração do tratamento para doenças respiratórias, como a asma e a DPOC, ambas de prevalência expressiva na população mundial.^(^ 1 ^,^ 2 ^)^

A inserção desses dispositivos na rotina do tratamento das doenças respiratórias ocorreu na década de 1950, com a criação dos primeiros inaladores pressurizados (IPrs). Posteriormente, na década de 1990, foram desenvolvidos os inaladores de pó (IP).^(^ 3 ^-^ 5 ^)^ Os fármacos disponibilizados através desses dispositivos possuem ação broncodilatadora e/ou anti-inflamatória.^(^ 1 ^,^ 3 ^,^ 6 ^)^ Sua deposição diretamente no órgão alvo apresenta vantagens, como a redução de efeitos sistêmicos adversos e a rápida ação na redução dos sintomas.^(^ 4 ^,^ 6 ^,^ 7 ^)^

Cada tipo de dispositivo inalatório possui particularidades para sua correta utilização. A escolha do inalador a ser prescrito depende de uma série de fatores, que vão desde a familiaridade do paciente com a técnica de uso de determinado dispositivo e o grau de deposição nas vias aéreas, passando por relação custo/benefício, portabilidade e cognição do paciente.^(^ 8 ^-^ 11 ^)^

Um dos fatores documentados como importante contribuinte na falha do controle de asma e DPOC, resultando em aumento na procura por atendimentos de emergência e hospitalizações evitáveis, é a técnica incorreta de utilização dos dispositivos inalatórios.^(^ 12 ^,^ 13 ^)^

Portanto, o presente estudo teve como objetivo de conhecer, através de um check-list dos passos para a realização da técnica inalatória, os principais erros cometidos e o perfil dos indivíduos que apresentam maior dificuldade ao utilizar inaladores.

## Métodos

O presente estudo transversal, observacional, aberto e não controlado fez parte de uma pesquisa transversal, de base populacional, realizada no ano de 2012, envolvendo 1.722 domicílios da cidade de Pelotas (RS) em indivíduos com idade ≥ 10 anos.^(^ 14 ^)^

Aqueles que referiram ter recebido o diagnóstico médico de asma, bronquite ou enfisema e que utilizavam dispositivos inalatórios de dose controlada foram convidados a participar do presente estudo, que foi realizado no período entre maio e setembro de 2012. Foi verificada a técnica de utilização dos dispositivos inalatórios e aplicado um pequeno questionário adicional à pesquisa principal, pelo qual foram coletados aspectos referentes ao uso do inalador. Aqueles que utilizavam o dispositivo com o auxílio de outras pessoas ou utilizavam espaçadores com máscara para o uso de IPr foram excluídos pois não atenderiam aos passos presentes no check-list. 

As variáveis coletadas na pesquisa principal que foram também analisadas no presente estudo foram sexo, idade, escolaridade (em anos de estudo) e nível socioeconômico, através do uso do Indicador Econômico Nacional (IEN) categorizado em tercis. Foi questionado quem havia indicado o uso do inalador (médicos pneumologistas, clínicos gerais, outros especialistas ou leigos), se houve a realização da demonstração da técnica de inalação pelo médico no momento da prescrição do medicamento (sim/não), a frequência do uso do inalador (crônico ou somente em exacerbações) e a forma de aquisição do medicamento (farmácia comum, Programa Farmácia Popular,^(^ 15 ^)^ rede pública ou outros).

A técnica de inalação foi verificada com base em um check-list para cada modelo de inalador, que foi elaborado através das recomendações do III Consenso Brasileiro no Manejo da Asma para a utilização de dispositivos inalatórios.^(^ 8 ^)^ O preenchimento do questionário e a verificação da técnica foram realizados no domicílio do participante por duas entrevistadoras treinadas. Para a observação da técnica era solicitado ao usuário do inalador que fizesse uma demonstração de como procedia no momento de usar o medicamento com seu próprio inalador ou com um placebo fornecido no momento da entrevista. Após o preenchimento do check-list*,* o indivíduo recebia uma orientação sobre os passos que necessitavam ser corrigidos. Os dados foram posteriormente digitados em duplicata através do programa EpiData, versão 3.1 (EpiData Association, Odense, Dinamarca), e realizada a checagem de inconsistências.

O desempenho da técnica foi avaliado de diferentes maneiras: 1) fases principais da inalação: pré-inalação (compreendendo o preparo da dose), inalação (desde exalar o ar antes da inspiração até a inalação do medicamento) e pós-inalação (pausa inspiratória ao final da inspiração e repetição do procedimento caso indicado). Essas informações permitiram a criação de variáveis dicotômicas; para cada fase plenamente cumprida, a mesma era categorizada em "sim/não", conforme particularidades de cada inalador; 2) presença de pelo menos um erro nos itens do check-list, categorizado como "sim/não" e 3) proporção de erros cometidos nos itens do check-list*, *o que originou uma variável contínua. Essas duas últimas abordagens compreenderam desde o momento de exalação do ar antes da inalação até a pausa inspiratória ao final da inspiração. 

Os resultados foram descritos tendo os inaladores como unidade de análise, pois cada inalador possui características particulares de uso, mesmo quando utilizados por um mesmo indivíduo. Foram utilizadas frequências absolutas e relativas e seus IC95%. As análises bivariadas foram realizadas através do teste do qui-quadrado de heterogeneidade e do teste de tendência linear para os desfechos dicotomizados, assim como o teste de Kruskal-Wallis para os desfechos numéricos com distribuição assimétrica. Foi utilizado o pacote estatístico STATA, versão 12.0 (StataCorp LP, College Station, TX, EUA).

Assim como na pesquisa principal, o projeto foi aprovado pelo Comitê de Ética da Faculdade de Medicina da Universidade Federal de Pelotas em 1º de dezembro de 2011, sob o número 77/11, e os participantes do subestudo ou seus responsáveis assinaram um termo de consentimento livre e esclarecido para a presente etapa da pesquisa. 

## Resultados

A Figura 1 descreve a amostra do estudo principal e do subestudo. A amostra do subestudo foi composta por 110 indivíduos que aceitaram realizar a verificação da técnica inalatória. Foram utilizados 143 inaladores, sendo 94 IPrs e 49 IP. Dos 146 usuários de inaladores detectados na pesquisa principal, 21 (14,4%) recusaram a participação ou não foram localizados durante a realização do subestudo, 8 (5,5%) foram excluídos por não desempenharem a técnica de inalação sem auxílio, e 7 (4,8%) foram excluídos por utilizarem espaçadores com máscara.

**Figura 1:** Fluxograma da composição da amostra.

Dos 49 IP utilizados, 40 eram modelos de inalador com cápsula, 7 eram do tipo Diskus, e 2 eram do tipo Turbuhaler. Outros modelos não foram utilizados entre os participantes do estudo, exceto o modelo Respimat, utilizado por 3 indivíduos. Esse modelo foi excluído das análises por suas particularidades na técnica de uso e pelo baixo número de observações.

A forma de aquisição da maioria dos inaladores foi através da farmácia comum (77%), seguido da aquisição através da rede pública (11%) e através do Programa Farmácia Popular (10%). O restante dos inaladores foi adquirido de outras formas, como recebimento de doações ou por ação judicial.

Quanto aos erros cometidos durante a técnica de inalação, tanto para os IPrs como para os IP, os mais frequentes foram não expirar antes da inalação (cometido por mais da metade da amostra total) e não fazer a pausa inspiratória após a inalação (Tabela 1). Na verificação da técnica de uso do IPr, também se destacam o não agitar o dispositivo e não aguardar 15-30 s para nova inalação (entre aqueles que faziam uso de mais de uma dose). Todos os erros apresentados nos itens do check-list estiveram presentes em maior proporção na faixa etária de 60 anos ou mais, exceto o fato de não se expirar antes da inalação do IPr, que apresentou relativa homogeneidade entre todas as categorias de idade. 

**Tabela 1:** Descrição dos erros, por passos, da técnica de utilização de inaladores pressurizados e inaladores de pó (N = 143 inaladores) no total da amostra e por faixa etária.a

Passo da técnica de inalação	Erros no total da amostra	Erros por faixa etária		
		10-19 anos	20-59 anos	60 anos ou mais
Inalador pressurizado	(n = 94)	(n = 24)	(n = 61)	(n = 9)
Não agitar o dispositivo	20 (21,3)	6 (25,0)	11 (18,0)	3 (33,3)
Não posicionar a saída do bocal verticalmente a 4-5 cm da boca ou entre os lábios	3 (3,2)	1 (4,2)	2 (3,3)	0 (0,0)
Não manter a boca aberta	2 (2,1)	1 (4,2)	1 (1,6)	0 (0,0)
Não expirar normalmente	62 (66,0)	16 (66,7)	41 (67,2)	5 (66,0)
Não acionar o dispositivo no início da inspiração lenta e profunda	7 (7,5)	1 (4,2)	4 (6,6)	2 (22,2)
Não fazer pausa pós-inspiratória de, no mínimo, 10 segundos	27 (28,7)	7 (29,2)	16 (26,2)	4 (44,4)
Não aguardar por 15-30 segundos para um novo acionamento^b^	45 (57,7)	11 (55,0)	26 (54,2)	8 (88,9)
Inalador de pó	(n = 49)	(n = 4)	(n = 29)	(n = 16)
Erro no preparo da dose (todos os modelos)	3 (6,1)	0 (0,0)	0 (0,0)	3 (18,8)
Não expirar normalmente	23 (46,9)	1 (25,0)	12 (41,4)	10 (62,5)
Não colocar o dispositivo na boca	1 (2,0)	0 (0,0)	0 (0,0)	1 (6,3)
Não inspirar o mais rápido e profundo possível	3 (6,1)	0 (0,0)	0 (0,0)	3 (18,7)
Não fazer pausa pós-inspiratória de 10 segundos	12 (24,5)	0 (0,0)	7 (24,1)	5 (31,3)
Se de dose única, não realizar nova inspiração, mais profunda que a anterior, se restar pó na cápsula^c^	7 (17,5)	1 (25,0)	2 (8,7)	4 (30,7)

a Valores expressos em n (%). bn = 78. cn = 40.

A Tabela 2 mostra a descrição dos inaladores da amostra segundo características sociodemográficas e relativas à prescrição do medicamento, bem como a presença de erros na técnica de utilização dos inaladores conforme essas características. Entre os usuários de IPr e de IP, 72% e 51%, respectivamente, cometeram algum erro durante a inalação dos medicamentos. A proporção média de erros nos itens do check-list foi de 21%. Percebe-se que aqueles com menor escolaridade (até 8 anos de estudo) cometeram mais erros no uso dos dois tipos de inaladores em relação aos mais escolarizados. Quanto ao nível socioeconômico, também se observou diferenças significativas entre os tercis do IEN para a presença de algum erro na técnica de uso do IPr e a proporção média de erros, sendo essas médias maiores entre os menos favorecidos economicamente. As diferenças na execução da técnica de inalação entre as demais variáveis não apresentaram diferenças com significância estatística.

**Tabela 2:** Descrição da utilização dos inaladores, segundo variáveis demográficas, socioeconômicas, fonte de indicação, demonstração da técnica e frequência de uso (N = 143 inaladores).

Variáveis	Inalador pressurizado			Inalador de pó			Percentual médio de erros (EP)^†^	p
	n (%)	% algum erro na técnica (IC95%)	p	n (%)	% algum erro na técnica (IC95%)	p		
Sexo								
Masculino	29 (30,8)	65,5 (47,7-83,4)	0,323	16 (32,6)	43,8 (18,0-69,5)	0,478	19,1 (21,5)	0,379
Feminino	65 (69,2)	75,4 (64,7-86,1)		33 (67,4)	54,5 (36,8-72,2)		21,8 (20,0)	
Idade, anos								
10-19	24 (25,5)	79,2 (62,4-96,0)	0,376*	4 (8,2)	25,0 (0,0-75,3)	0,219*	19,5 (17,7)	0,399
20-39	32 (34,0)	71,9 (55,8-87,9)		10 (20,4)	50,0 (16,5-83,5)		17,5 (15,0)	
40-59	29 (30,9)	69,0 (51,6-86,3)		19 (38,8)	47,4 (23,7-71,0)		21,3 (22,2)	
60 ou mais	9 (9,6)	66,7 (33,5-99,8)		16 (32,7)	62,5 (37,4-87,6)		27,8 (26,4)	
Escolaridade, anos								
0-8	46 (48,9)	84,8 (74,1-95,4)	0,018*	16 (32,7)	81,3 (61,0-100,0)	0,010*	27,5 (18,0)	< 0,001
9-11	28 (29,8)	60,7 (42,0-79,4)		16 (32,7)	37,5 (12,4-62,6)		16,7 (21,2)	
12 ou mais	20 (21,3)	60,0 (37,7-82,3)		17 (34,7)	35,3 (11,3-59,3)		15,0 (20,6)	
IEN, tercis^a^								
1 (mais pobres)	25 (26,9)	88,0 (74,8-100,0)	0,044*	11 (22,5)	72,7 (44,4-100,0)	0,080*	27,2 (17,4)	0,017
2	38 (40,9)	71,1 (56,2-85,9)		22 (44,9)	50,0 (28,1-71,9)		21,3 (22,0)	
3 (mais ricos)	30 (32,3)	63,3 (45,6-81,1)		16 (32,7)	37,5 (12,4-62,6)		16,0 (19,5)	
Indicação de uso do inalador								
Pneumologista	35 (37,2)	60,0 (43,3-76,7)	0,090	35 (72,9)	44,4 (27,6-61,3)	0,125	17,3 (20,8)	0,073
Clinico geral/outra especialidade	49 (52,1)	81,6 (70,5-92,7)		13 (27,1)	69,2 (42,4-96,0)		25,3 (20,0)	
Não médico	10 (10,6)	70,0 (39,7-100,0)		0 (0,0)	-		20,0 (16,3)	
Recebeu demonstração do médico								
Sim	52 (55,3)	69,2 (56,4-82,1)	0,375	36 (73,5)	43,2 (26,6-59,8)	0,056	18,4 (20,3)	0,112
Não	32 (34,0)	78,1 (63,4-92,9)		13 (26,5)	75,0 (48,7-100,0)		26,3 (21,0)	
Frequência de uso								
Crônico	23 (24,5)	69,6 (50,1-89,0)	0,732	31 (63,3)	54,8 (36,5-73,1)	0,483	23,0 (21,3)	0,216
Crises	71 (75,5)	73,2 (62,7-83,7)		18 (36,7)	44,4 (20,2-68,7)		19,7 (19,9)	
TOTAL	94 (100)	72,4 (63,1-81,6)		49 (100)	51,0 (36,5-65,5)		20,9 (20,4)	

IEN: Indicador Econômico Nacional

a n = 142. Valores de p determinados pelo teste do qui-quadrado de heterogeneidade, exceto onde indicado

* Teste do qui-quadrado de tendência linear

† Teste de Kruskal-Wallis.

A Figura 2 mostra um diagrama de Venn para a técnica de uso do inalador nas três fases: pré-inalação, inalação e pós-inalação. Em apenas 13% das demonstrações os inaladores foram utilizados de forma plenamente correta, sendo que em 11% das demonstrações da técnica houve erros em todas as fases durante a realização do check-list. 

**Figura 2:** Diagrama de Venn mostrando proporções de demonstrações corretas conforme as fases de uso de inaladores (N = 143 inaladores). Fase 1: pré-inalação, com o preparo da dose plenamente correto. Fase 2: expiração e aproximação do dispositivo para a boca até o final da inalação de forma plenamente correta. Fase 3: finalização da técnica de forma plenamente correta. Em 16 demonstrações (11%), não houve nenhuma fase plenamente correta.

## Discussão

O presente estudo descreve as dificuldades dos pacientes na utilização dos dispositivos inalatórios no tratamento para asma e DPOC. Por tratar-se de um estudo de base populacional, possibilita um reconhecimento do perfil geral dos usuários desse tipo de medicamento, sejam esses pacientes em acompanhamento nos serviços de saúde, sejam aqueles que não tiveram consultas médicas por um longo tempo, sejam até aqueles que tiveram o uso dos inaladores indicados por leigos. 

Uma limitação do presente estudo é que não foram avaliadas questões referentes a severidade/estadiamento das doenças respiratórias e conformidade da terapia inalatória recomendada, nem foram verificadas associações da mesma com o controle das pneumopatias, o que poderia reforçar a necessidade da utilização adequada da técnica. Também, devido ao tamanho de amostra limitado, não houve poder estatístico para detectar algumas associações entre a realização da técnica e determinadas características dos indivíduos. Somente foi possível detectar diferenças significativas (p ≤ 0,05) para as razões de prevalência iguais ou superiores a 1,5. 

Outro possível fator limitador que pode estar presente nos resultados é o viés de informação. Os usuários de inaladores, apesar de não terem sido informados com antecedência de que teriam sua técnica verificada, podem tê-la realizado mais corretamente por estarem sendo observados, havendo uma distinção da sua realização rotineira da técnica com redução do número de erros detectados. Também não foi possível identificar objetivamente se o fluxo de inspiração no uso do IP obteve a velocidade mínima preconizada para os diferentes dispositivos inalatórios.

A proporção da presença de algum erro durante a realização da técnica seguindo o check-list, em relação ao número de inaladores testados, foi menor que a esperada de acordo com a literatura: em um estudo prévio, embora os pacientes referissem conhecer a técnica adequada dos inaladores, aproximadamente 90% cometeram algum erro.^(^ 16 ^)^ Além disso, em uma pesquisa realizada por inquérito telefônico, 77 dos 87 entrevistados afirmaram que sua técnica nunca havia sido verificada por um profissional da saúde, e, dos 26 eleitos para uma demonstração, nenhum obteve desempenho satisfatório.^(^ 17 ^)^ Já um estudo realizado no estado da Bahia detectou que mais da metade dos indivíduos estudados apresentavam uma boa técnica inalatória para todos os modelos de inalador; porém, a amostra foi composta por indivíduos que recebiam acompanhamento e verificação da técnica periodicamente, e o critério de classificação do desempenho da técnica considerou como bom desempenho aqueles com 75% de acertos ou mais.^(^ 18 ^)^

As características dos pacientes com indicação de uso de inaladores também são um ponto importante de atenção no momento da prescrição dos mesmos. Em estudos prévios, foi detectado que idosos cometem mais erros por apresentarem alterações cognitivas, entre outros fatores.^(^ 12 ^,^ 19 ^,^ 20 ^)^ Em nosso estudo, houve uma maior proporção de erros na faixa etária de maiores de 60 anos; porém, não foi possível detectar uma diferença significativa na técnica de inalação em relação às faixas etárias menores. A faixa etária de maiores de 60 anos foi a menor em nossa amostra, possivelmente sendo esse o fator que impediu a detecção de diferenças significativas em relação à técnica utilizada por aqueles mais jovens. Uma explicação para o baixo número de participantes nessa faixa etária mais idosa seria o número menor de usuários de IPrs, mesmo esse sendo um grupo com um número expressivo de pessoas que referem possuir doenças respiratórias. ^(^ 14 ^)^ Muitos podem não ter se adaptado a esse tipo de medicamento e preferirem utilizar a nebulização, que está indicada àqueles que não possuem condições cognitivas para o uso de outros métodos de medicação inalatória^(^ 1 ^,^ 2 ^)^; além disso, um critério de exclusão do presente estudo era a utilização dos inaladores com a ajuda de outra pessoa ou o uso de IPr com espaçadores com máscara, recursos frequentemente utilizados nessa faixa etária. Segundo um estudo prévio,^(^ 21 ^)^ os usuários de nebulizadores possuem idade avançada, condições respiratórias mais graves e grandes dificuldades no uso de IPrs.

Nossos resultados mostraram uma tendência linear significativa para uma maior frequência de erros entre aqueles de menor nível socioeconômico (exceto para IP, com p > 0,05, mas com tendência na mesma direção) e menor escolaridade. Tais resultados vão ao encontro de achados anteriores. ^(^ 12 ^,^ 19 ^)^ A escolaridade e o nível socioeconômico são indicadores semelhantes e mostram a importância do nível educacional não apenas como facilitador da compreensão da técnica em si, mas também sobre o conhecimento da doença,^(^ 13 ^)^ melhorando de maneira geral a adesão ao tratamento. Tais achados sugerem a necessidade de implementação de medidas educativas em relação à doença e à técnica de uso dos medicamentos, especialmente entre aqueles menos favorecidos socioeconomicamente.

Os erros mais comuns descritos na literatura sobre o uso dos dispositivos inalatórios são os seguintes: não exalar antes da inspiração e não manter a apneia após a inalação, posição incorreta do inalador e falha ao executar uma inspiração profunda com alto fluxo.^(^ 16 ^,^ 22 ^)^ Em nosso estudo, a maioria desses erros estava presente de maneira expressiva.

Houve um maior número de erros na realização da técnica com o IPr do que com o IP. Esse fato tem sido documentado na literatura, como em um estudo,^(^ 23 ^)^ no qual houve uma maior proporção de indivíduos que cometeram pelo menos um erro na técnica, assim como de erros considerados de maior importância para a efetividade do tratamento, no uso dos IPrs em comparação com o uso dos IP. 

Muitos indivíduos também não agitam o IPr antes de utilizá-lo. No momento da realização da pesquisa, a elaboração do check-list da técnica foi baseada em um consenso brasileiro,^(^ 8 ^)^ e esse passo ainda era considerado obrigatório para todos os IPrs. Porém, conforme diretrizes mais recentes, dependendo de sua formulação (solução ou suspensão), nem todos os IPrs necessitam ser agitados sempre^(^ 1 ^)^; entretanto, a maioria das recomendações quanto à técnica mantém a orientação quanto à necessidade de se agitar o dispositivo antes de seu uso,^(^ 24 ^)^ uma vez que é sabido que o não cumprimento dessa etapa pode reduzir em até 36% o aproveitamento da dose do aerossol.^(^ 25 ^)^

Outra diferença da técnica de uso dos IPrs daquele consenso^(^ 8 ^)^ com as diretrizes atuais para o manejo da asma^(^ 1 ^)^ foi a remoção do clorofluorcarbono das formulações e substituição pelo hidrofluoralcano (HFA). Houve assim uma facilitação da técnica - o HFA propicia um jato um jato de maior duração, reduzindo a necessidade de uma coordenação fina entre o disparo e a inalação do medicamento.^(^ 1 ^)^ Apesar de problemas na sincronia entre o disparo e a inalação serem bastante documentados,^(^ 25 ^)^ problemas nesse passo foram pouco detectados, provavelmente pelos critérios de elegibilidade para a participação no estudo.

Outro erro documentado na literatura é a inadequada distância do IPr em relação aos lábios do paciente^(^ 16 ^)^; porém, não contabilizamos como erro o fato de muitas pessoas colocarem o inalador entre os lábios no momento da inalação. Nas diretrizes mais recentes, é aceito que a técnica inalatória seja realizada dessa maneira, por reduzir a evaporação de gás, aumentando a deposição pulmonar e reduzindo o risco de disparo na região perioral.^(^ 1 ^)^

Outra falha na técnica detectada em nosso estudo, assim como presente em estudos anteriores,^(^ 16 ^,^ 22 ^)^ é o fato de o indivíduo não exalar antes da inalação. Segundo um artigo de revisão objetivando principalmente a importância desse passo, é recomendado que seja realizada uma exalação não forçada até a capacidade residual funcional ou o volume residual, otimizando a efetividade da técnica inalatória.^(^ 26 ^)^ Realizar uma pausa inspiratória de 10 segundos também está descrita nas recomendações para uma ótima deposição pulmonar do medicamento para todos os modelos de dispositivos inalatórios. Porém, para o IPr, não é mais preconizado que se aguarde 30 segundos para a utilização de mais de uma dose, como no consenso utilizado para a elaboração de nosso check-list.^(^ 1 ^,^ 8 ^,^ 24 ^)^

Em nosso estudo, as maiores proporções de erros ocorreram entre os indivíduos que não receberam uma demonstração no momento da prescrição do medicamento quando comparados com aqueles que receberam uma demonstração do uso correto dos inaladores, apesar de esse resultado não ter atingido diferença estatisticamente significativa. Esse fator já foi previamente documentado como colaborador para a realização de uma técnica insatisfatória.^(^ 27 ^)^ Deve-se salientar que muitos profissionais da área da saúde não têm um conhecimento adequado sobre o uso dos IP e IPrs, colaborando para a alta proporção de pessoas que usam erroneamente seus medicamentos inalatórios, mesmo quando tenham recebido uma demonstração do uso dos inaladores em algum momento.^(^ 28 ^)^ É válido ressaltar que, além do treinamento no momento da prescrição, os pacientes devem receber orientações periódicas ao longo do tratamento, pois a técnica correta costuma ser esquecida com o passar do tempo.^(^ 22 ^,^ 23 ^)^

Em uma intervenção realizada por farmacêuticos na Alemanha, 757 pacientes com diagnóstico de asma ou DPOC tiveram sua técnica observada e registrada através de um check-list, sendo feitas orientações e correções das falhas no primeiro encontro. Aproximadamente 80% apresentaram algum erro ao início do estudo. Após um mês da avaliação inicial, uma nova demonstração foi solicitada, e essa proporção caiu para 28,3%. ^(^ 29 ^)^ Assim, atividades educacionais, mesmo que pontuais, podem ser benéficas aos usuários de inaladores.

A orientação dos pacientes e de seus cuidadores, realizada pelos profissionais da área da saúde, tem um papel fundamental na utilização dos inaladores a fim de diminuir os erros e otimizar a terapêutica.^(^ 9 ^,^ 17 ^,^ 22 ^,^ 26 ^)^ A técnica inadequada constitui-se em um fator de risco para o mau controle das doenças respiratórias,^(^ 12 ^,^ 13 ^)^ estando associada à má adesão ao tratamento. ^(^ 30 ^)^ Destaca-se, no entanto, que esse é um fator de risco modificável, sendo que alguns aspectos esclarecidos no presente estudo podem funcionar como pontos de referência a serem reforçados na técnica inalatória, além de permitir identificar o perfil daqueles pacientes que potencialmente necessitarão de maiores esclarecimentos sobre o uso desses medicamentos.

Concluímos, portanto, que os principais erros cometidos pelos pacientes ao utilizarem inaladores são não expirar antes da inalação e não realizar a pausa inspiratória após a inalação, para ambos os modelos de inalador. Merecem especial atenção aqueles pacientes menos favorecidos socioeconomicamente, pois apresentam um maior risco de realizar a técnica inadequada no uso dos IP, assim como aqueles de faixas etárias mais avançadas, por apresentarem uma maior proporção de erros na realização da técnica.
